# Alpha and beta diversity of functional traits in subtropical evergreen broad-leaved secondary forest communities

**DOI:** 10.3389/fpls.2024.1223351

**Published:** 2024-04-23

**Authors:** Liangjin Yao, Chuping Wu, Zhigao Wang, Bo Jiang

**Affiliations:** ^1^Zhejiang Academy of Forestry, Hangzhou, China; ^2^Zhejiang Hangzhou Urban Forest Ecosystem Research Station, Hangzhou, China

**Keywords:** functional traits, intra-specific variation, environmental filtering, dispersal limitation, community assembly functional traits, community assembly

## Abstract

**Introduction:**

Intra-speciic variation is the main source of functional trait diversity and has similar ecological effects as inter-speciic variation.

**Methods:**

We studied 79 species and 3546 individuals from 50 ixed monitoring plots in subtropical evergreen broad - leaved secondary forests in Zhejiang Province, China. Using trait gradient analysis, we examined nine traits (speciic leaf area, leaf dry matter content, wood density, leaf area, chlorophyll content, leaf nitrogen content, leaf phosphorus content, leaf potassium content, and nitrogen-phosphorus ratio) by decomposing species functional traits into alpha (within-community) and beta (among-communities) measure the impact of environmental gradients and the presence of other species on the variation of traits.

**Result:**

All nine functional traits showed some degree of differentiation in the forest communities, with a greater range of variation in alpha values than in beta values . Correlations were signiicantly different between the trait differences in the communities. The alpha values of each trait showed a higher correlation with other components than the beta values. The factors affecting intra-speciic trait variation were relatively complex. The alpha component had a more signiicant and stronger effect on intra-speciic trait variation compared to the beta component. Abiotic factors, such as soil nutrient content, soil nitrogen-phosphorus content, directly affected the beta component. In contrast, biotic factors, such as tree height variation, had a direct and stronger effect on the alpha component.

**Discussion:**

Our results demonstrate that alpha and beta components, as independent differentiation axes among coexisting species, have different sensitivities to different environmental factors and traits in different ecological strategies and spatial scales. Trait gradient analysis can more clearly reveal the variation patterns of species traits in communities, which will help to understand the scale effects and potential mechanisms of trait relationships.

## 1 Introduction

Forest communities rely on competition and allocation of resources such as space, time, and biotic factors, which are crucial in driving species coexistence and ecological niche differentiation (Lamprecht et al., 2018; Midolo et al., 2019; Staude et al., 2022). However, solely considering inter-specific trait variation may underestimate the overlap of species’ ecological niches and functional traits, potentially biasing our understanding of species coexistence, community function, and dynamics (Violle et al., 2007). Growing evidence suggests that variation in traits within the species is more likely to determine the competitive interactions of individual species and community-level responses to global environmental change (Bassar et al., 2017; Henn et al., 2018; Midolo et al., 2019; Thomas et al., 2020; Rixen et al., 2022; Staude et al., 2022).

Several studies have found that intra-specific trait variation explains a substantial proportion of the overall variation in traits within and between communities, having strong predictive power in predicting ecological processes, community-building mechanisms, and ecosystem function (Violle et al., 2012). Siefert et al. (Siefert et al., 2015) found that intra-specific trait variation explained 25% of the total trait variation within a community and 32% of the trait variation between communities. Albert et al. (Albert et al., 2010) found that nearly 30% of trait variation came from intra-specific variation, with the highest intra-specific variation in leaf nitrogen and carbon content. Intra-specific trait variation has been observed to facilitate the coexistence of species utilizing both biotic and abiotic filtering mechanisms. (Jung et al., 2010) and can help plants cope with environmental changes (Laforest-Lapointe et al., 2014). Studies of the communities of grassland found on the Qinghai-Tibet Plateau also found that intra-specific trait variation dominated the functional diversity changes in response to inter-annual climate fluctuations, buffering the impacts of climate on community stability. Intra-specific trait variation also influences the diversity of functional traits and the components of functional diversity, including alpha and beta diversity. (Bello et al., 2017; Thomas et al., 2020) The presence of intra-specific variation can also influence community building by affecting the distribution pattern of functional trait beta diversity which has implications for nutrient cycling, crop disease resistance, and community building (Cornwell et al., 2006; Lecerf and Chauvet, 2008; Garrett et al., 2011; Thomas et al., 2020).

Species that are screened in a local environment are theoretically able to adapt to the small habitat, which results in functional convergence among species within the community (de Bello et al., 2021). However, interactions among the biotic community lead to competition and exclusion of similar species, known as limiting similarity, which limits the similarity among coexisting species. Both individual environmental filtering and limiting similarity contribute to community structure and maintain biodiversity (Violle et al., 2012). Trait gradient analysis (TGA) provides a better understanding of interactions among communities at different levels (Cornwell et al., 2006). intra-specific trait variation can be divided into alpha and beta components. The alpha component represents the differences between the mean attributes of a species and the community’s mean attributes at its location, reflecting differences in the adaptive strategies of species that coexist within the same community environment (Swenson, 2013; Yao et al., 2020b). The beta component represents a species’ position on this trait gradient, reflecting the strength of the response signal of functional traits among species along environmental gradients. This helps to explain the respective contributions of dispersal limitation and environmental filtering in the process of community assembly (Yao et al., 2020a, Yao et al., 2020b). By studying the functional similarity between alpha and beta components within species (Swenson, 2013), it is possible to directly reveal whether species coexistence is due to strong environmental filtering or processes such as limiting similarity.

Subtropical evergreen broad-leaved forests are highly diverse and play critical roles in maintaining biodiversity and ecosystem functions. Although facing habitat loss and threats from human activities, the species composition, structure, and function of subtropical evergreen secondary forests are significant for biodiversity conservation, climate regulation, soil and water conservation, and prevention of land degradation (Zhang et al., 2022). To gain insights into species’ functional traits and community dynamics, we conducted a study of 50 fixed monitoring plots of subtropical evergreen secondary forests in Zhejiang Province, with 79 species and 3546 individuals. We collected data on nine functional traits and community data, and the trait gradient method to decompose species functional traits into alpha traits (within the community) and beta traits (between communities) to quantify the effects of environmental gradients and coexisting species on trait variation. We hypothesized that small habitat heterogeneity and few individuals of the same species at the local scale would result in high similarity among coexisting individuals and smaller alpha diversity within species, while large habitat heterogeneity among different plots would result in larger beta diversity within species due to significant differences between individuals of the identical species that are growing in separate plots.

## 2 Study area and methods

### 2.1 Overview of the study area

Wuchao Mountain National Forest Park is situated in Xianlin Town, Yuhang District, Hangzhou City, Zhejiang Province, China (33.410^◦^ N, 120.013^◦^ E), and is part of the veins of Tianmu Mountain. This park is a natural forest ecosystem situated in the suburban area of the city, covering a total area of 522 hectares. It has an average altitude of 264 meters, with the highest point reaching 495 meters. Positioned within the central subtropical zone, the park benefits from abundant water resources and favorable heat conditions. The average annual temperature is 16.1°C, with January being the coldest month (averaging 3.6°C) and August being the warmest (averaging 38.4°C). The park also experiences an annual average of 1970.6 sunshine hours and has a plant growth period of 311 days, and the forest coverage rate is approximately 93%, showcasing its rich plant species diversity. The area is mainly characterized by red and yellow soil types, and evergreen broad-leaved forests are the predominant vegetation, featuring trees such as *Cyclobalanopsis glauca*, *Castanopsis chinensis*, *Schima superba*, *Castanopsis sclerophylla*. The park has undergone significant restoration efforts, as it was previously subjected to human interference before the 1970s. For the last four decades, the forest has been entirely protected and designated for afforestation purposes.

### 2.2 Data collection and analysis

Between October 2020 and June 2021, we established fifty permanent forest dynamics plots, each covering 0.04 ha, in Wuchao Mountain National Nature Reserve. The plots were located at elevations ranging from 340.1 to 467.4 m, where the forest composition, structure, and habitat were all homogeneous, which the tree species composition within the studied plots is similar. In every plot, the diameter at breast height (DBH) and height (H) of all woody stems with a DBH of 1 cm or greater were measured and identified to the species level in collaboration with local botanists.

We evaluated key functional traits including specific leaf area (SLA, mm^2^/mg), leaf dry matter content (LDMC, mg/m^2^), relative chlorophyll content (SPAD), wood density (WD, g/cm³), leaf nitrogen content (LNC, mg/g), leaf phosphorus content (LPC, mg/g), leaf potassium content (LKC, mg/g) and leaf nitrogen to phosphorus ratio (N:P) separately To obtain these measurements, we collected 10-20 healthy and mature sun leaves for each species and analyzed their morphology, chlorophyll content, and nitrogen to phosphorus content. We used a leaf area meter, an electronic balance, and an oven to measure specific leaf area and leaf dry matter content while a handheld chlorophyll meter (Konica Minolta SPAD-502) was used to measure leaf chlorophyll content. Leaf elemental analysis was conducted in the laboratory on dried leaf samples. In each of the permanent forest dynamics plots, we gathered branches and leaves from all individuals with a DBH of 10 cm or greater. Concerning individuals with a DBH less than 10 cm, we selected the five largest individuals for each species. If in a plot, fewer than five individuals of a species were found, we sampled all individuals of that species present in the plot. The functional trait information we measured from the all the individual level in all the plots. In total, we measured and sampled 3546 individuals, representing 79 species across the 50 plots.

### 2.3 Trait gradient analysis

The community was organized based on the average trait values of the species, with the trait values weighted by species abundance. The functional traits were categorized into two values: alpha and beta traits. The beta trait indicates the species’ position on the trait gradient, representing its response to environmental changes within the community. On the other hand, the alpha trait refers to the disparity between the average species properties and the community averages at their respective positions. This reflects the diverse adaptation strategies of coexisting species to the shared community environment (Cornwell et al., 2006). These calculations were performed using the formulas proposed by Cornwell (Cornwell et al., 2006).


(1) $$\bar{p_{j}}{=}_{\frac{\sum_{i=1}^{S}aij\sum ti}{\sum_{i=1}^{S}aij}}$$

(2) $$\beta_{i}=\frac{\sum_{j=1}^{P}\bar{pj}aij}{\sum_{j=1}^{P}aij}$$

(3) $$t_{i}=\alpha_{i}+\beta_{i}$$

The process of Trait Gradient Analysis (TGA) involves breaking down traits into various components. Firstly, the abundance of each species in a given sample is denoted by aij. The average trait of a sample, pj, is then calculated by weighing the abundance of each species in that sample (Equation (1)). All samples are then arranged in order of their average trait (p>_j_), thus creating the trait gradient. The value of the beta trait within a given species (i) is determined by calculating the average trait of that species across all samples, with each value being weighted by the abundance of that species (Equation (2)). The average range of a given species (i) is referred to as the ti value. Finally, the discrepancy between the average range (t_i_) and the beta trait (as per Equation (3)) is known as the alpha trait value of species i.

### 2.4 Analysis method

In this study, we employed a range of statistical methods to analyze the functional traits of different species. First, to analyze the functional traits and examine the variation between alpha and beta values, we employed the TGA method (Yao et al., 2020b). Additionally, we utilized the skewness analysis method to investigate the distribution of alpha and beta values, determining if the distribution exhibited right-skewness, left-skewness, symmetry, or uniformity. To compare functional traits among species, we adopted chi-square tests (*p< 0.05; **p< 0.01; ***p< 0.001) and transformed the SLA, LA, LNC, LKC, and N:P values using a log10 transformation, as these traits did not follow a normal distribution. To identify the main influencing factors, we conducted separate principal component analyses on the alpha component, beta component, soil nutrients, and biotic factors. Subsequently, we employed structural equation modeling (SEM) to investigate the direct and indirect impacts of soil nutrients and biotic factors on intra-specific trait variation, taking into account the alpha and beta components. The SEM was fitted using the maximum likelihood method, and the goodness of fit was evaluated based on several metrics, including the comparative fit index (CFI), root mean square error of approximation (RMSEA), significance probability value (P), and Akaike’s information criterion (AIC). A good fit was indicated by CFI > 0.9, RMSEA< 0.08, P > 0.05, and a smaller AIC value. Statistical analysis and plotting were performed in R 4.2.1.

## 3 Result

### 3.1 Functional trait variation in evergreen broad-leaved secondary forest communities

The evergreen broad-leaved secondary forest community displays some degree of functional trait differentiation among its nine functional traits. Plant traits such as SLA, LA, and CHL have the highest mean values, whereas LDMC and LKC have the lowest mean values. LPC and LA exhibit the greatest range of variation in mean values, while CHL and WD exhibit the least. Additionally, the scope of alpha values is greater than the beta values, with LPC and LKC having the highest range of variation in alpha values, and CHL and WD having the lowest. LPC and LA exhibit the largest range of variation in beta values, while CHL and N:P exhibit the smallest range of variation. Ecologically, LA and LPC possess the broadest niche width, whereas CHL, WD, and N:P exhibit the narrowest niche width. The functional traits with the largest mean values in the plot are SLA, CHL, and LA, while LDMC and LPC have the smallest mean values. The functional traits with the largest range of variation in plot mean values are LPC and LA, while CHL and WD have the smallest range of variation (**Table 1**).

**Table 1:** Mean and range of plant functional traits parameters.

Parameter	Species characteristics									Plot characteristics		
Traits	T_i, mean_	_Ti_	_Range of Ti_	_beta_	_Range of beta_	_alpha_	_Range of alpha_	_Rs,_ _mean_	_Rs_	_Pj, mean_	_Pj, min–max_	_Range of Pj_
SLA	2.21	1.71	0.79	2.08	0.19	-0.45	0.84	0.07	0.00	2.13	2.08	0.19
		2.50		2.27		0.39			0.19		2.27	
LDMC	0.43	0.14	0.98	0.34	0.13	-0.20	0.85	0.06	0.00	0.41	0.34	0.15
		1.12		0.47		0.65			0.15		0.49	
WD	0.97	0.66	0.48	0.93	0.13	-0.35	0.46	0.05	0.00	1.02	0.92	0.14
		1.14		1.06		0.11			0.13		1.06	
LA	1.76	1.01	1.19	1.39	0.58	-0.46	0.85	0.25	0.00	1.66	1.39	0.58
		2.20		1.97		0.39			0.58		1.97	
CHL	1.74	1.49	0.38	1.72	0.09	-0.27	0.36	0.04	0.00	1.76	1.71	0.11
		1.87		1.81		0.09			0.10		1.82	
LNC	1.23	0.81	0.86	1.12	0.24	-0.36	0.75	0.08	0.00	1.16	1.11	0.25
		1.67		1.36		0.39			0.25		1.36	
LPC	0.96	0.14	1.37	0.62	0.58	-0.28	0.97	0.20	0.00	0.75	0.61	0.59
		1.51		1.20		0.69			0.58		1.20	
LKC	0.95	0.29	1.17	0.86	0.14	-0.65	1.19	0.06	0.00	0.91	0.85	0.15
		1.46		1.00		0.54			0.14		1.00	
N:P	1.28	0.75	0.7	1.24	0.11	-0.54	0.7	0.05	0.00	1.31	1.24	0.15
		1.45		1.35		0.16			0.14		1.39	

T_i_, the discrepancy between the average range of all samples. p_j_, the average trait of a sample. Rs, the niche breadth.

### 3.2 Functional trait differentiation and correlation of evergreen broad-leaved secondary forest community

**Figure 1:**
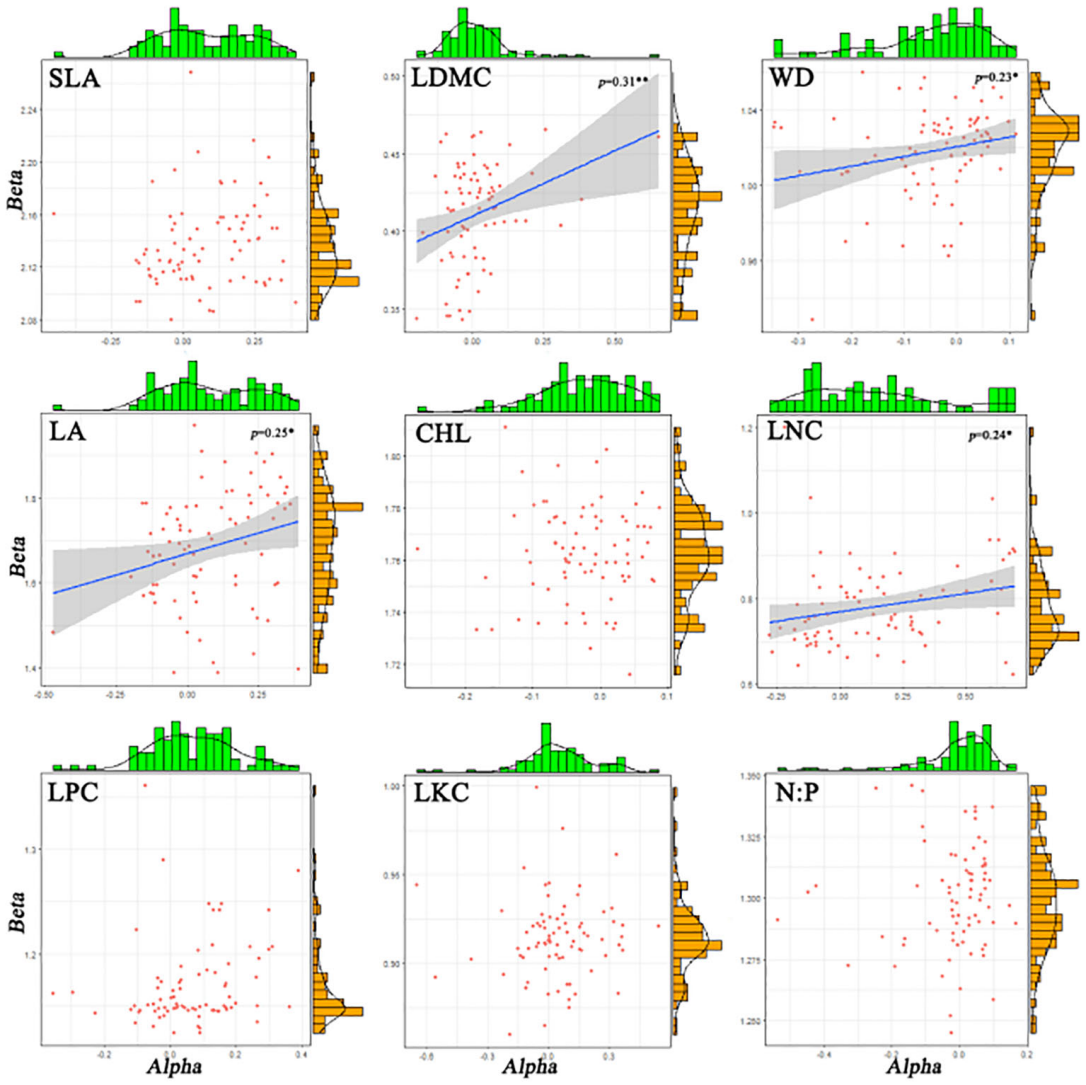
The histograms and distribution plot of alpha and beta components for functional traits of evergreen broad-leaved secondary forest communities. Solid blue lines indicate linear regression fits and the surrounding grey-shaded area denotes the 95% condifence interval. *R*^2^ refers to the goodness of fit of the model, the higher the fit.

**Figure 2:**
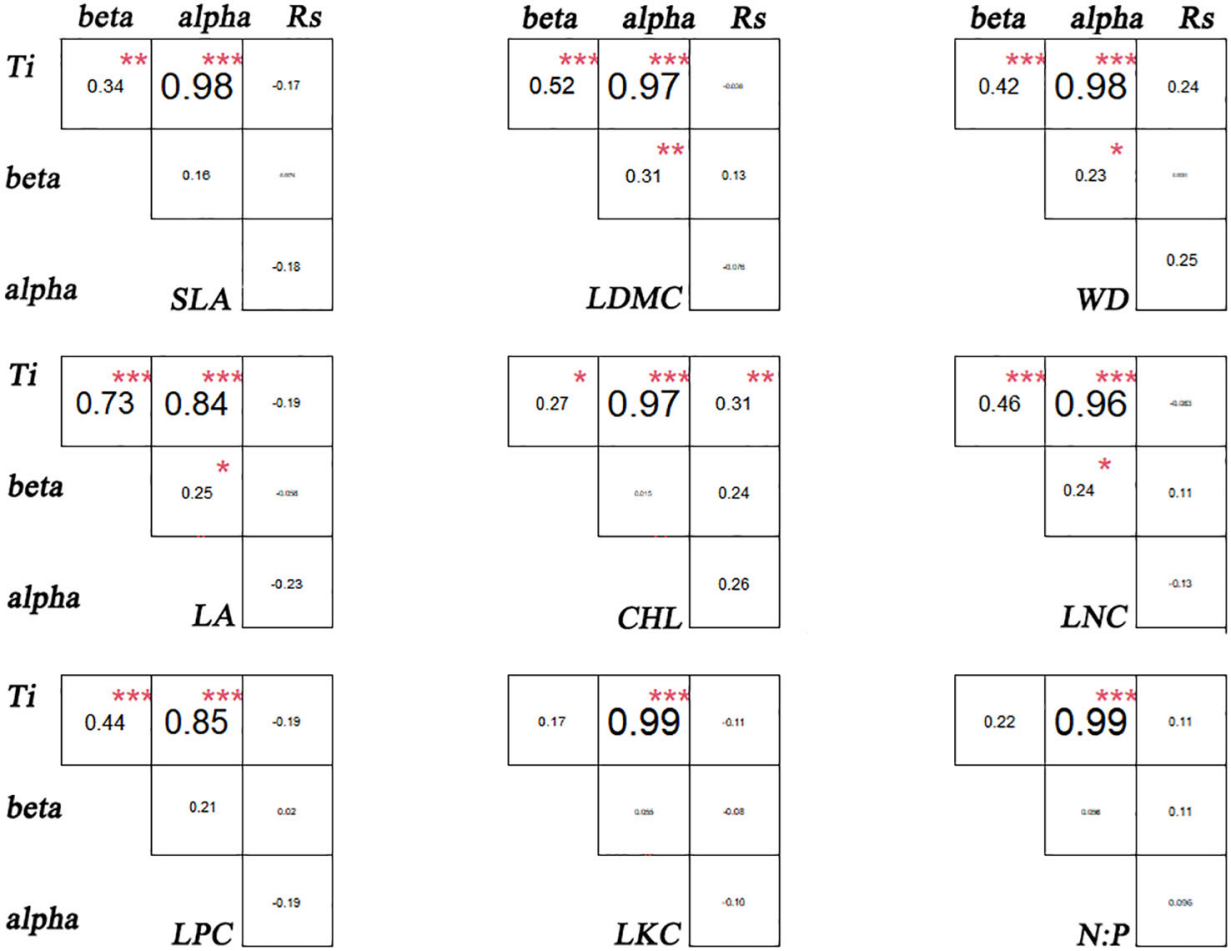
Association of functional traits in evergreen broad-leaved secondary forest communities. * p< 0.05; ** p< 0.01; *** p< 0.001. Significant correlation.

There are significant differences in the correlations among different functional traits in the evergreen broad-leaved secondary forest community. There is a strong correlation (p< 0.001, r > 0.84) between T_i_ (the mean value of functional traits for each species) and alpha for all traits except LKC and N: P. There is a weak significant correlation (p< 0.001, r > 0.84) between T_i_ and beta for all traits except LKC and N: P. It is hardly found that there is a significant correlation between T_i_ and R_s_ (species richness). Only LDMC, WD, LA, and LNC show weak significant correlations between alpha and beta (p< 0.05, r > 0.24), while there is no significant correlation between other functional traits. The significant correlation (p > 0.05) between R_s_ and the differentiation of functional traits (T_i_, alpha and beta) for each trait cannot be found. We found that intra-specific trait variation dominates among species trait variations (**Figures 1**, **2**).

### 3.3 Factors affecting intra-specific trait variation in evergreen broad-leaved secondary forests

**Figure 3:**
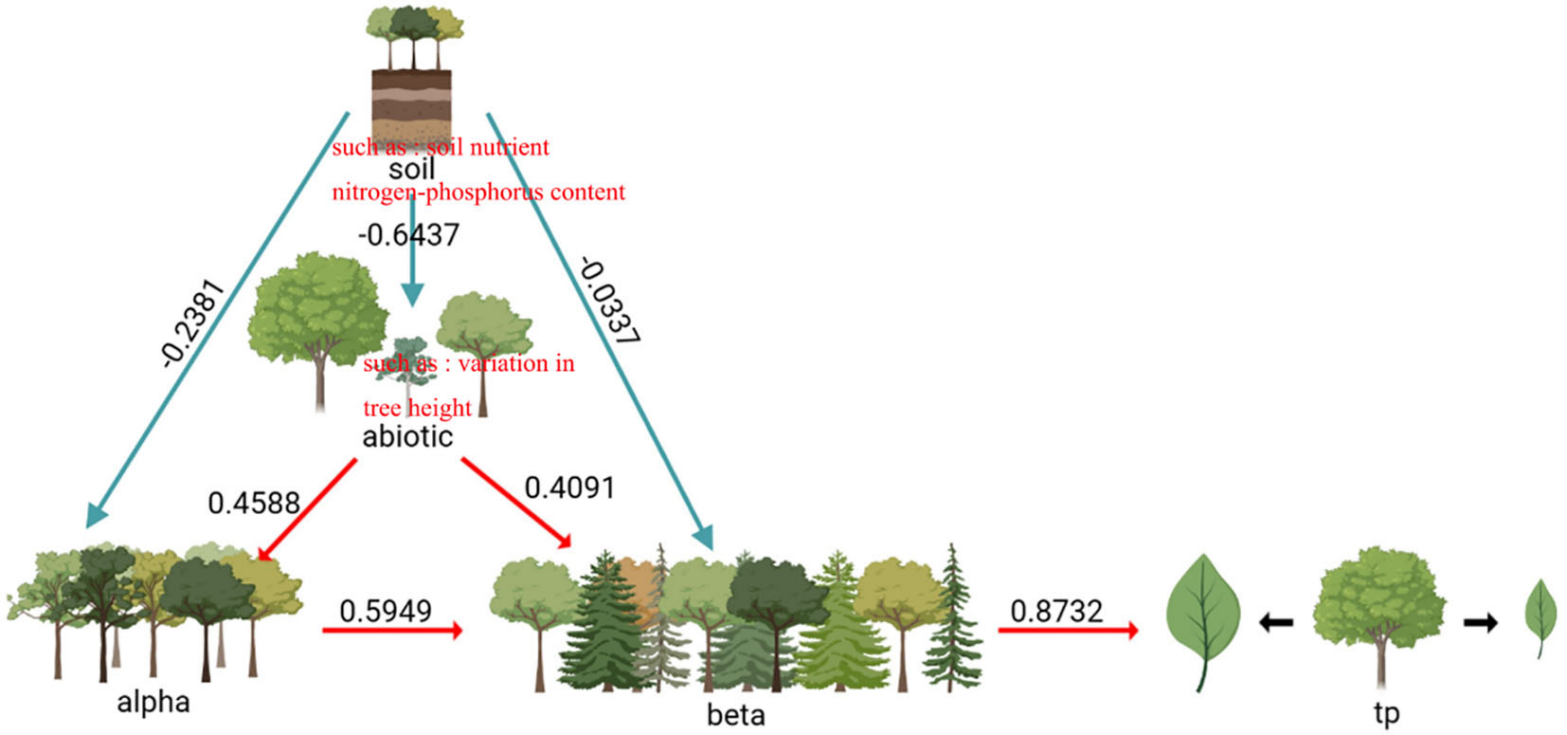
Effect of biotic and soil factors, alpha components, and beta components on intraspecific trait variation. Path coefficients negative χ2 = 5.62, df=7, p=0.78 positive RMSE=0.00, AIC=33.50.

The factors influencing the variation of intra-specific traits in the evergreen broad-leaved secondary forests of Wuchao Mountain in Zhejiang are relatively complex. As shown in **Figure 3**, the variables explain 37% of the observed variation. The direct impact of the alpha and beta components on intra-specific trait variation was found to be statistically significant (P< 0.05), and the path coefficients were respectively 0.58 and 0.19. Abiotic factors, such as soil nutrients and nitrogen-phosphorus content, have a direct effect on the beta component (P< 0.001, path coefficient of 0.72). Biotic factors, such as variation in tree height, have a direct effect on the alpha component (P< 0.001, path coefficient of 0.46). Abiotic factors have a direct effect on biotic factors (P< 0.001, path coefficient of 0.28). Overall, these results demonstrate that multiple factors contribute to intra-specific trait variation in the evergreen broad-leaved secondary forests of Wuchao Mountain in Zhejiang, with both biotic and abiotic factors playing a crucial role.

## 4 Discussion

### 4.1 Functional trait components of evergreen broad-leaved secondary forest communities

The variation observed in plant functional traits indicates the capacity of species to adapt to changes in their environment and is closely linked to community composition and ecological processes, like productivity and litter decomposition (Orwin et al., 2010). Our study found varying degrees of differentiation in the alpha and beta components of nine functional traits in the evergreen broad-leaved secondary forest community. This implies that the differences in the extent of variation in each trait or differences in trait combinations reflect the response of each functional trait to environmental change and species coexistence, ultimately leading to the differentiation of species’ ecological niches and ecological strategies. However, the degree of response of different traits varies (Yao et al., 2021) and may be attributed to the allometric relationships between different traits, resulting in inconsistent trait stability. The highest degree of differentiation in alpha components was leaf area. This is likely because leaves are the link through which plants convert and utilize energy through photosynthesis, making them most sensitive to habitat changes (Markesteijn et al., 2011).

Further research confirms that some plant functional traits, including leaf traits and plant nutrient content, exhibit stronger plasticity in local environments and competition (Kunstler et al., 2016; Thomas et al., 2020). Leaf dry matter content and wood density are relatively stable and exhibit a delayed reaction to changes in the environment, as they are long-term tree growth results that are related to the plant’s support, resistance to pest and disease erosion, and resource acquisition abilities (Choat et al., 2012). However, the range of trait changes across species is larger, possibly because of differences in survival strategies formed by different species adapting to different environments. Both evergreen and deciduous tree species are major dominant species in the secondary forest community of Wuchao Mountain (Yao et al., 2022), and different survival strategies affect trait variation. Conservative species may show lower within-species trait variation while displaying absolute trait values (Rixen et al., 2022). For example, evergreen tree species increase their light resource utilization efficiency through high leaf dry matter content (positive alpha value) and low specific leaf area (negative alpha value), while deciduous tree species acquire light resources rapidly during the period of active growth by having a high specific leaf area (positive alpha value) and low dry matter content (negative alpha value). Our study also confirms the existence of obvious trait differentiation phenomena, such as leaf phosphorus content, leaf dry matter content, and specific leaf area in the subtropical evergreen broad-leaved secondary forest community.

Based on Yao et al (Yao et al., 2021), the alpha components of 9 functional traits in the secondary evergreen broad-leaved forest community on Wuchao Mountain exhibit a greater range of variation than the beta components. This suggests that the interactions among coexisting species within each sample community have a stronger influence than the effects of environmental differences between communities. The small geographic area of the study, which consisted of 30 randomly located fixed plots within mid-stage forest communities that had recovered from disturbance, may be the main reason for this observation. Larger trees are more likely to experience competitive exclusion due to their greater demand for habitat resources. Species dispersal limitations and interactions generally occur at small community scales, while environmental filtering is more prevalent at larger community scales. Additionally, the sample plots in the secondary evergreen broad-leaved forest community on Wuchao Mountain exhibit a larger ecological niche width compared to individual species. This may be attributed to the increased environmental heterogeneity within the community, which expands the ecological niche of the species and increases the niche width of the sample plots. These findings carry significant implications for comprehending the interactions and species distribution patterns of the ecosystem in this region.

### 4.2 Association of functional trait components in evergreen broad-leaved secondary forest communities

Plants’ ability to adapt to the heterogeneous biotic and abiotic environments within a community is enhanced by the interactions between their functional trait components. These interactions deepen the connections between species, communities, and ecosystems (Kraft and Ackerly, 2010; Kunstler et al., 2016). Plant functional trait values are determined by the combined effects of alpha and beta values, and species ecological strategies are determined by the multidimensional trait combinations. The interactions between multidimensional trait combinations demonstrate that species utilize similar ways to adapt to environmental filtering in a community. Each trait independently defines a functional axis in species strategy, and due to the interactions between coexisting species, independent trait axes may differentiate and couple showing high correlations at both global and regional scales but might be uncorrelated at local scales (Webb et al., 2002; Cornwell et al., 2006). It was shown in the studies that the correlations between alpha components are stronger than those between beta components, indicating that multiple plant functional traits exhibit high overall convergent adaptability to the same biotic factor as competition. This may be owing to the small sampling area, low quantity of individuals within the plot, small environmental heterogeneity, and higher genetic correlations among adjacent individuals at the local scale (Albert et al., 2010). This proves that biotic factors, like competition, led to the convergence of functional traits in species adaptation, and natural selection or environmental stress increases trait differentiation at the local scale (Kunstler et al., 2016). The mid-successional evergreen broad-leaved forest in Wuchao Mountain selectively filters out evergreen and deciduous tree species through inter-specific competition. Species adapt to intra-specific or inter-specific competition within the community by adopting similar ways, such as through the plasticity of one or more functional traits (Diaz et al., 2004).

### 4.3 Factors influencing intra-specific trait variation in evergreen broad-leaved secondary forests

The evolution of species traits is driven by two main factors: limiting similarity and environmental filtering. SEM found that Biotic competition significantly increases trait variation within species in the secondary broad-leaved evergreen forest of Wuchao Mountain. This finding confirms that species interactions and dispersal limitations mainly occur at the small community scale, while environmental filtering occurs at the larger community scale (Thomas et al., 2020). Our study focuses on a local region, and existing research suggests that at relatively small scales (<100m), species turnover determines the pattern of beta diversity in the formation of subtropical suburban secondary evergreen broad-leaved forest. At small scales, the main influence is diffusion limitation, with increasing environmental filtering effects as the scale expands, and a gradual decrease in the influence of diffusion limitation (Yao et al., 2023). Therefore, environmental filtering is the primary factor influencing community assembly in the studied area. As coexisting species interact more, the differences between the alpha traits of species and the community level increase, indicating stronger differentiation of species’ alpha traits and a stronger correlation between species’ alpha traits. In a community, species use different combinations of niche differentiation and trait variation to adapt to their environment and reduce competition for resources. The functional trait beta component is also influenced significantly by environmental and spatial variables. Because all plants use similar strategies to utilize resources such as nitrogen, phosphorus, potassium, water, light, and CO_2_ in the local environment, species niche differentiation is not very evident when there is limited availability of resources in the habitat. At the regional scale, habitat heterogeneity among different sample sites is large, and individuals belonging to different sample sites may have significantly different genetic structures (Albert et al., 2010). Thus, at the community level, environmental filtering reduces within-species variation, while niche differentiation increases it.

The functional traits influence the distribution of plants along environmental gradients, which also reflect their interactions with coexisting species. Studies have found that trait gradient analysis integrates two different perspectives. Firstly, it emphasizes the role of resource differentiation, interference, and trait variation among coexisting species (referred to as the alpha value) to understand the mechanisms of biodiversity maintenance (Weiher and Keddy, 1995). Secondly, the other highlights the impact of environmental heterogeneity on species coexistence by integrating changes along environmental gradients such as soil, climate, and topography (known as beta value) (Wright, 2002). Kooyman et al. (Kooyman et al., 2010) concluded that differences in species traits are essential in determining community stability in similar environmental conditions, indicating that the differences in species traits are an important factor in determining community stability in communities with similar environmental conditions. Intra-specific trait variation can also impact the outcome of species competition, as shown by Flöder et al. (Flöder et al., 2021) and Kunstler et al (Kunstler et al., 2016). Habitat filtering plays a crucial role in trait variation, particularly in conditions where environmental gradients differ significantly, such as along latitudinal gradients with marked climate differences. For example, a study on the functional trait differences of plants across North America found that trait differences were strongly correlated with environmental factors, but the impact of species competition intensity on trait variation was small (Šímová et al., 2015).

## 5 Conclusion

This study used the trait gradient method to analyze the functional traits of species into alpha (within-community) and beta (between-community) components, revealing trait variation patterns in communities. The basic driving forces behind species coexistence in local communities are the opposing forces of environmental filtering and similarity limitation. We discovered that biotic interactions within plots had a greater impact on trait variation in the evergreen broad-leaved secondary forest community of Wuchao Mountain, while abiotic factors had a relatively weaker influence. In small habitats with limited resources and relatively homogenous conditions, dominant competitive exclusion among species played a significant role in community assembly, with limiting similarity being a key factor. The findings of this research will contribute to the comprehension of the fundamental connections between species traits, thus enhancing the comprehension of species coexistence mechanisms and clarifying the instability of evergreen broad-leaved secondary forests during the succession process.

## Data availability statement

The original contributions presented in the study are included in the article/supplementary material. Further inquiries can be directed to the corresponding author.

## Author contributions

LY and BJ designed this study and improved the English language and grammatical editing. LY wrote the first draft of the manuscript and performed the data analysis. ZW and CW did the fieldwork. BJ gave guidance and methodological advice. All the coauthors contributed to the discussion, revision, and improvement of the manuscript. All authors have read and agreed to the published version of the manuscript.
