# Supplementary figures and images for: Alpha and beta diversity of functional traits in subtropical evergreen broad-leaved secondary forest communities
Source: Front Plant Sci. 2024 Apr 23;15:1223351. doi: 10.3389/fpls.2024.1223351 (PMC11075155; doi:10.3389/fpls.2024.1223351)

Supplementary


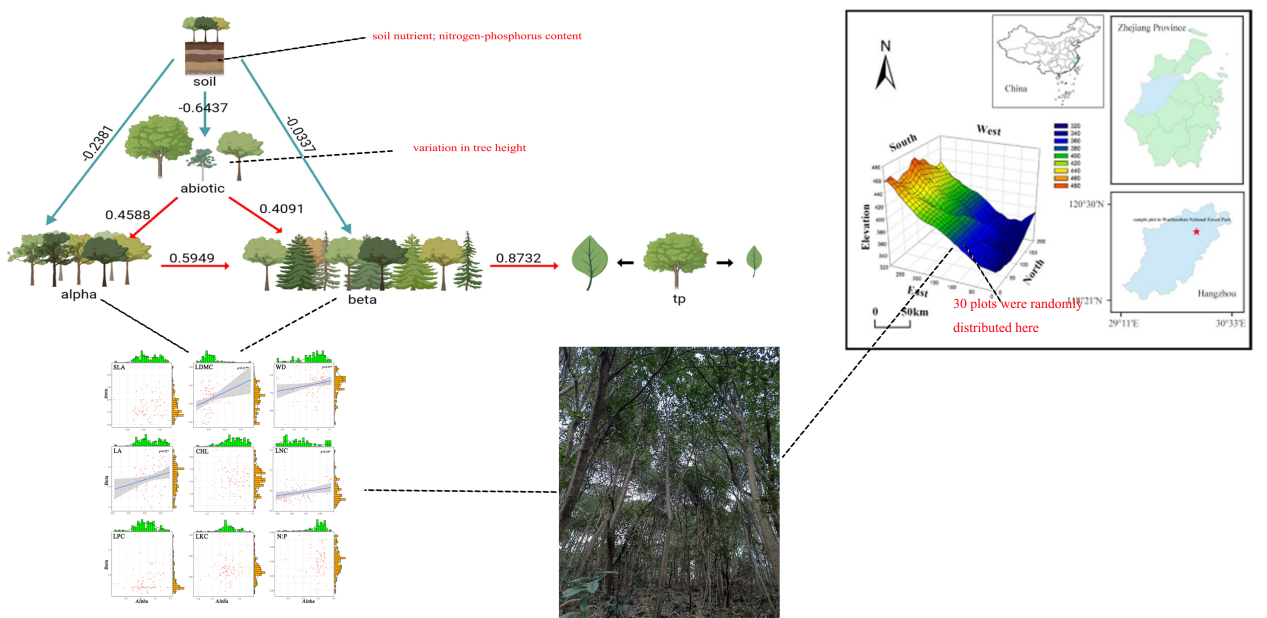

Supplement: Supplementary file 1 [file DataSheet_1.docx]
